# Effect of Budesonide Oral Suspension on Time to First Dysphagia Symptom Response and Dysphagia Symptom Resolution Outcomes in Patients With Eosinophilic Esophagitis

**DOI:** 10.1111/jgh.70205

**Published:** 2026-02-19

**Authors:** Evan S. Dellon, David A. Katzka, Vincent A. Mukkada, Gary W. Falk, Muna J. Tahir, P. Cristian Gugiu, Jessamyn Blau, Brian Terreri

**Affiliations:** ^1^ Center for Esophageal Diseases and Swallowing, Division of Gastroenterology and Hepatology, Department of Medicine University of North Carolina at Chapel Hill Chapel Hill North Carolina USA; ^2^ Division of Digestive and Liver Diseases NewYork‐Presbyterian/Columbia University Irving Medical Center New York New York USA; ^3^ Division of Gastroenterology, Hepatology and Nutrition Cincinnati Children's Hospital Medical Center, University of Cincinnati College of Medicine Cincinnati Ohio USA; ^4^ Division of Gastroenterology and Hepatology Perelman School of Medicine, University of Pennsylvania Philadelphia Pennsylvania USA; ^5^ Takeda Development Center Americas, Inc. Cambridge Massachusetts USA; ^6^ Takeda Pharmaceuticals USA, Inc. Cambridge Massachusetts USA; ^7^ Takeda Pharmaceuticals USA, Inc. Lexington Massachusetts USA

**Keywords:** budesonide oral suspension, DSQ, dysphagia‐free days, eosinophilic esophagitis, swallowed corticosteroid

## Abstract

**Background and Aim:**

We examined post hoc the effect of budesonide oral suspension (BOS/Eohilia) 2.0 mg twice daily (b.i.d.) on dysphagia symptom outcomes in patients with eosinophilic esophagitis (EoE).

**Methods:**

Patients aged 11–55 years who received BOS 2.0 mg b.i.d. or placebo during phase 2 or phase 3, 12‐week, double‐blind, placebo‐controlled clinical trials (MPI 101‐06 and SHP621‐301) were included. The Kaplan–Meier method captured time to first dysphagia symptom response (≥ 30% reduction in the Dysphagia Symptom Questionnaire [DSQ] score from baseline). Efficacy outcomes using the DSQ at weeks 4, 8, and 12 of therapy were: the proportion of patients with complete dysphagia symptom resolution; change from baseline in the number of dysphagia‐free days; and the proportion of patients with dysphagia symptoms, but without adaptive behaviors or pain. Each outcome was measured based on daily DSQ records in the 2 weeks before each study visit.

**Results:**

Overall, 411 patients from MPI 101‐06 (BOS, *n* = 51; placebo, *n* = 42) and SHP621‐301 (BOS, *n* = 213; placebo, *n* = 105) were included. Median time to first dysphagia symptom response was significantly shorter for BOS‐ than placebo‐treated patients (MPI 101‐06, *p* = 0.0239; SHP621‐301, *p* = 0.0156), with separation between groups at week 2, the earliest time point measured. At all time points, higher proportions of BOS‐ than placebo‐treated patients had complete dysphagia symptom resolution or had dysphagia symptoms but no adaptive behaviors or pain. BOS‐ versus placebo‐treated patients also had greater improvements from baseline in the number of dysphagia‐free days.

**Conclusions:**

BOS is efficacious in managing dysphagia in EoE across several symptom metrics.

**ClinicalTrials.gov:** NCT01642212; NCT02605837.

## Graphical abstract

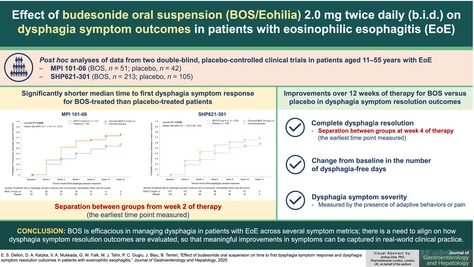

## 1 Introduction

Eosinophilic esophagitis (EoE) is a chronic, immune‐mediated disease, characterized clinically by esophageal dysfunction, including dysphagia [1, 2, 3], and eosinophilic inflammation (≥ 15 eosinophils per high‐power field) [1, 3, 4, 5]. Dysphagia is the most commonly reported symptom in adolescents and adults [3]. There is a well‐established discordance between symptoms and histologic activity in EoE [6]. Clinical trials therefore typically examine both histologic and symptom outcomes, as per guidance from the US Food and Drug Administration (FDA) for drug development in EoE [7], but may also capture other metrics such as endoscopic severity and health‐related quality of life [7, 8, 9, 10, 11, 12].

The Dysphagia Symptom Questionnaire (DSQ) is a four‐item, patient‐reported outcome (PRO) measure that is specifically designed to assess the frequency and severity of dysphagia and pain with swallowing in patients with EoE [13, 14, 15]. The DSQ has been validated in adolescents and adults aged ≥ 11 years with EoE [13, 16] and has been used as a primary efficacy outcome in clinical trials to measure dysphagia based on the absolute or percentage change in the DSQ score from baseline [8, 9, 10, 13]. However, this change can be difficult to interpret because the score represents both the frequency and severity of dysphagia [6]. Given the importance that patients with EoE place on achieving an improvement in dysphagia symptoms [17], there is a growing interest in measuring complete dysphagia symptom resolution and dysphagia‐free days in clinical trials for EoE to capture clinically meaningful indicators of disease activity following treatment [6].

Budesonide oral suspension (BOS) 2.0 mg twice daily (b.i.d.) is an FDA‐approved swallowed corticosteroid indicated for 12‐week use in patients with EoE aged ≥ 11 years [18]. During two 12‐week, placebo‐controlled clinical trials in patients with EoE and dysphagia (MPI 101‐06 and SHP621‐301), BOS‐treated patients demonstrated significantly greater improvements in DSQ scores, as well as in histologic and endoscopic outcomes, from baseline to week 12 of therapy than placebo‐treated patients [8, 9].

In these post hoc analyses, we aimed to examine the effect of BOS 2.0 mg b.i.d. versus placebo on the time to first dysphagia symptom response and on dysphagia symptom resolution outcomes in patients with EoE who received up to 12 weeks of therapy during MPI 101‐06 or SHP621‐301.

## 2 Methods

### 2.1 Study Design and Population

MPI 101‐06 (phase 2; NCT01642212) and SHP621‐301 (phase 3; NCT02605837) were multicenter, randomized, double‐blind, placebo‐controlled clinical trials in patients aged 11–55 years with EoE and dysphagia who received up to 12 weeks of BOS 2.0 mg b.i.d. (Eohilia) or placebo [8, 9]. These post hoc analyses also included one patient aged 9 years (MPI 101‐06); however, BOS is only approved by the FDA for patients aged ≥ 11 years [18]. All patients provided written informed consent/assent before enrollment, and both studies were performed in accordance with the Declaration of Helsinki, the Good Clinical Practice Guidelines, and reported as per the Consolidated Standards of Reporting Trials statement [5, 8, 9]. Key inclusion and exclusion criteria are detailed in the Supporting Information; full criteria are described elsewhere [8, 9].

### 2.2 Post Hoc Analyses of Efficacy Outcomes

The DSQ specifically measures dysphagia in patients with EoE and uses a daily recall period [13]. Question (Q)1 asked whether patients had eaten solid foods; patients only completed the subsequent questions if they answered “Yes”. Q2 of the DSQ measures whether patients had experienced dysphagia (i.e., frequency of dysphagia). Q3 of the DSQ captures whether patients needed to take measures to aid swallowing or to gain relief during the most difficult period of dysphagia on a given day and what these measures were. Higher scores for Q3 indicate the need for greater adaptive behaviors to manage the patient's EoE and therefore may suggest greater EoE severity [14, 15]. Q4 is a standalone item that captures whether patients experienced pain when swallowing food. Further details of the DSQ can be found in Supporting Information and Table 1.

**TABLE 1:** Clinical concepts and attributes evaluated by the DSQ [13].

Clinical concepts	Symptom frequency (Q2 of the DSQ)	Symptom severity/adaptive behavior and pain (Q3 and Q4 of the DSQ)
Food went down slowly or got stuck in throat	X	
Got better or cleared up on its own		X
Drank liquid		X
Coughed and/or gagged		X
Vomited		X
Sought medical attention		X
Pain when swallowing food		X

*Note:* The scoring algorithm for the DSQ is constructed using responses from Q2 and Q3 (frequency and severity of dysphagia, respectively). Q4 (pain) is a standalone item that is scored separately.

Abbreviations: DSQ, Dysphagia Symptom Questionnaire; Q, question.

Time from baseline to the onset of first dysphagia symptom response (≥ 30% reduction in the DSQ score from baseline) was measured at 2‐week intervals during each of the 12‐week clinical trials. Efficacy outcomes assessed at weeks 4, 8, and 12 of therapy were the proportion of patients who reported complete dysphagia symptom resolution (defined as no symptoms [“No” to Q2 for each daily DSQ diary entry] in the 2 weeks before each study visit); the least‐squares (LS) mean (standard error of the mean) change from baseline in the number of dysphagia‐free days (number of days with “No” to Q2 of the DSQ in the 2 weeks before each study visit); and the proportion of patients who had dysphagia symptoms, but no adaptive behaviors or pain (“Yes” to Q2 of the DSQ for at least one daily diary entry in the 2 weeks before each study visit, but no daily score > 0 for Q3 or Q4). Data were also stratified by esophageal dilation history for these efficacy outcomes; further details can be found in Supporting Information.

Data were derived from DSQ scores if ≥ 8/14 days of DSQ diary entries in the 2 weeks before the study visit were non‐missing. If data for < 8/14 days were available, the score was set to missing. Patients who did not have efficacy data at a post‐baseline visit were classified as nonresponders at that visit.

### 2.3 Statistical Analyses

Outcomes were analyzed by treatment group using the intent‐to‐treat analysis set (MPI 101‐06) or full analysis set (SHP621‐301), which included all randomized patients who received at least one dose of BOS 2.0 mg b.i.d. or placebo.

For the time from baseline to the onset of first dysphagia symptom response, the Kaplan–Meier method was used. Hazard ratios were estimated using a Cox proportional hazards model adjusted for age group (< 18 or ≥ 18 years) and dietary restriction (no dietary restriction or dietary restriction). BOS and placebo treatment groups were compared using a stratified log‐rank test.

For binary outcomes at weeks 4, 8, and 12 of therapy, Cochran–Mantel–Haenszel (CMH)‐adjusted differences (Δ) in proportions of patients, with corresponding Newcombe 95% confidence intervals (CIs), were based on the CMH test stratified by age group and dietary restriction. If the Newcombe CI was not available, the CMH 95% CI was used.

LS mean treatment differences (Δ) and corresponding 95% CIs for the changes from baseline to weeks 4, 8, and 12 of therapy in the number of dysphagia‐free days were calculated using an analysis of covariance model, with treatment group, age group, and dietary restriction as factors, and baseline dysphagia‐free days as a continuous covariate.

## 3 Results

### 3.1 Patient Disposition and Baseline Demographics and Clinical Characteristics

Overall, 411 patients from MPI 101‐06 (BOS 2.0 mg b.i.d., *n* = 51; placebo, *n* = 42) and SHP621‐301 (BOS 2.0 mg b.i.d., *n* = 213; placebo, *n* = 105) were included in these post hoc analyses (Figure S1). Baseline demographics and clinical characteristics were generally similar between treatment groups in each clinical trial (Table 2) [8, 9].

**TABLE 2:** Baseline demographics and clinical characteristics for patients who received up to 12 weeks of BOS 2.0 mg b.i.d. or placebo in MPI 101‐06 or SHP621‐301 [8, 9] and were included in these post hoc analyses.

Demographic/clinical characteristic^a^	MPI 101‐06		SHP621‐301	
	BOS 2.0 mg b.i.d. (*n* = 51)	Placebo (*n* = 42)	BOS 2.0 mg b.i.d. (*n* = 213)	Placebo (*n* = 105)
Age, years, mean (SD)	22.3 (7.92)	20.8 (7.50)	33.8 (11.89)	33.9 (12.13)
Male, *n* (%)	35 (68.6)	29 (69.0)	129 (60.6)	62 (59.0)
Female, *n* (%)	16 (31.4)	13 (31.0)	84 (39.4)	43 (41.0)
Race, *n* (%)				
White	48 (94.1)	40 (95.2)	200 (93.9)	101 (96.2)
Black or African American	3 (5.9)	0 (0.0)	5 (2.3)	0 (0.0)
Native Hawaiian or Other Pacific Islander	0 (0.0)	0 (0.0)	0 (0.0)	1 (1.0)
American Indian or Alaska Native	0 (0.0)	0 (0.0)	1 (0.5)	0 (0.0)
Other^b^	0 (0.0)	2 (4.8)	7 (3.3)	3 (2.9)
DSQ score, mean (SD)^c^	30.8 (15.80)	29.0 (13.54)	30.3 (13.93)	30.4 (13.14)
Peak eosinophil count, eos/hpf, mean (SD)				
Overall	157.8 (96.08)	133.0 (81.55)	74.5 (39.16)	76.6 (45.04)
Proximal	100.9 (99.60)	53.4 (58.52)	39.4 (36.00)	43.2 (40.30)
Middle	103.8 (67.53)	94.4 (80.53)	55.8 (37.15)	58.1 (42.50)
Distal	107.4 (79.53)	95.6 (74.77)	56.4 (36.40)	56.0 (38.96)
Total EREFS, mean (SD)^d^	7.7 (3.54)	7.0 (3.31)	7.6 (3.55)	8.2 (3.25)
Prior esophageal dilation, *n* (%)	10 (19.6)	7 (16.7)	91 (42.7)	45 (42.9)
Concomitant medications, *n* (%)				
Inhaled/nasal corticosteroid	8 (15.7)	9 (21.4)	40 (18.8)	19 (18.1)
PPI	35 (68.6)	29 (69.0)	176 (82.6)	92 (87.6)

*Note:* SHP621‐301 table adapted from Hirano I, et al., *Clinical Gastroenterology and Hepatology* 20 (2022) 525–534; licensed under CC BY 4.0 (https://creativecommons.org/licenses/by/4.0/).

Abbreviations: b.i.d., twice daily; BOS, budesonide oral suspension; DSQ, Dysphagia Symptom Questionnaire; eos/hpf, eosinophils per high‐power field; EoE, eosinophilic esophagitis; EREFS, EoE Endoscopic Reference Score; PPI, proton pump inhibitor; SD, standard deviation.

^a^ Depending on individual demographics/clinical characteristics, *n* values may have varied. MPI 101–06: BOS, *n* = 48–51; placebo, *n* = 36–42; SHP621–301: BOS, *n* = 211–213; placebo, *n* = 103–105.

^b^ Other races were: Asian/Caucasian (*n* = 1); Black or African American, White (*n* = 2); Caucasian/African American (*n* = 1); Mexican (*n* = 2); Middle Eastern (*n* = 1); Mideastern (*n* = 1); Native American and Canadian Indian (*n* = 1); White and African American (*n* = 1); White and Black (*n* = 1); Not reported (*n* = 1).

^c^ DSQ scores were calculated from the combined scores of Q2 and Q3 from each daily diary completed during the 2 weeks before randomization. Scores were derived if ≥ 8/14 days of the DSQ diary were non‐missing. If data for < 8/14 days was available, the score was set to missing. Scores could theoretically range from 0 to 84.

^d^ Total EREFS was calculated by summing the scores for the five major feature categories for the proximal and distal esophagus: exudates or plaques (grade 0–2), fixed esophageal rings (grade 0–3), edema (grade 0–2), furrows (grade 0–2), and strictures (grade 0–1). Total scores could range from 0 to 20.

### 3.2 Post Hoc Efficacy Outcomes

#### 3.2.1 Time to First Dysphagia Symptom Response

**FIGURE 1:**
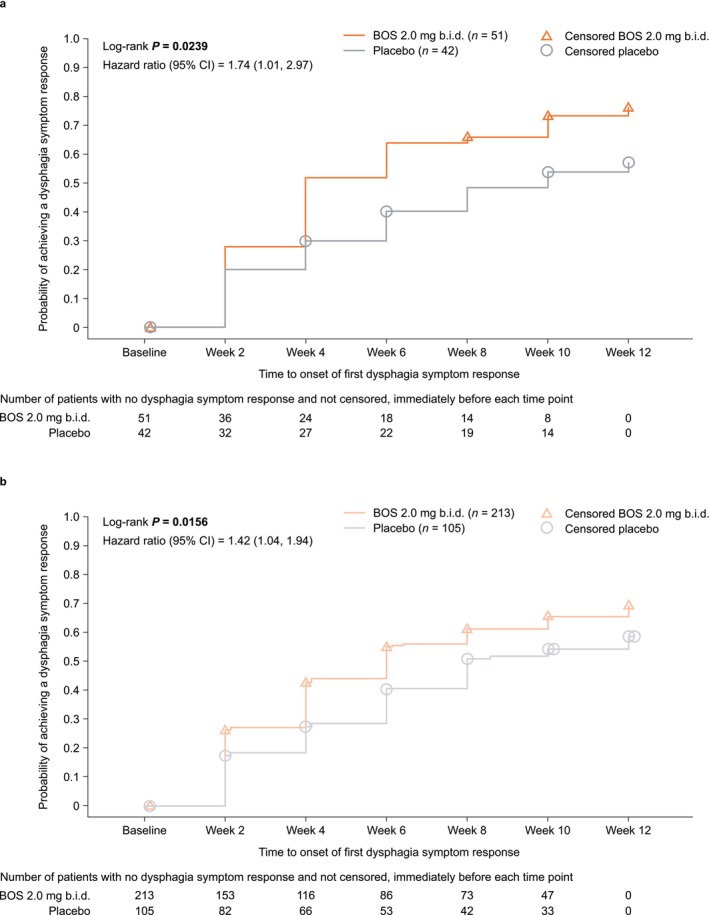
Kaplan–Meier plots of the time from baseline to first dysphagia symptom response (≥ 30% reduction in DSQ score from baseline) in patients who received up to 12 weeks of BOS 2.0 mg b.i.d. or placebo in (a) MPI 101‐06 or (b) SHP621‐301. Each 2‐week interval includes patients who either had a first response or were censored in that interval and excludes patients who previously had a response or were previously censored. Patients without post‐baseline DSQ assessments or with a missing baseline DSQ score were censored at day 1. A small number of patients were randomized 1 day before the start of treatment, resulting in some data points appearing outside of the 12‐week range. b.i.d., twice daily; BOS, budesonide oral suspension; CI, confidence interval; DSQ, Dysphagia Symptom Questionnaire. Adapted with permission from Wolters Kluwer Health Inc.: E. S. Dellon, D. A. Katzka, V. A. Mukkada, et al., “Effect of Budesonide Oral Suspension on Dysphagia Symptom Resolution and Time to First Dysphagia Symptom Response in Patients With Eosinophilic Esophagitis: Post Hoc Analysis of Phase 2 and Phase 3 Placebo‐Controlled Trials,” *American Journal of Gastroenterology* 119 (2024) S480; DOI: 10.14309/01.ajg.0001032140.06936.0e. https://journals.lww.com/ajg/pages/default.aspx.

The median time from baseline to the onset of first dysphagia symptom response (≥ 30% reduction in the DSQ score from baseline) was significantly shorter for BOS‐ than placebo‐treated patients (MPI 101‐06: 4.0 vs. 10.0 weeks, *p* = 0.0239; SHP621‐301: 6.0 vs. 8.0 weeks, *p* = 0.0156). Separation between treatment groups was observed as early as week 2 of therapy (the earliest time point measured) during both clinical trials (Figure 1).

#### 3.2.2 Complete Dysphagia Symptom Resolution

**FIGURE 2:**
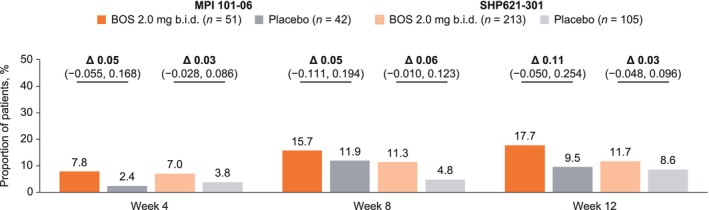
Proportion of patients who had complete dysphagia symptom resolution (“No” to Q2 for each daily DSQ diary entry in the 2 weeks before a study visit) at weeks 4, 8, and 12 of therapy with BOS 2.0 mg b.i.d. or placebo in MPI 101‐06 or SHP621‐301. Δ values (presented in decimal form) are CMH stratum‐adjusted treatment differences in proportions of patients, representing differences after making groups comparable (adjusting) for age and dietary restriction. These Δ values may therefore differ from the raw percentage differences that are calculable from the proportions of patients presented for each treatment group. Δ, CMH‐adjusted difference; b.i.d., twice daily; BOS, budesonide oral suspension; CI, confidence interval; CMH, Cochran–Mantel–Haenszel; DSQ, Dysphagia Symptom Questionnaire; Q, question.

At all time points assessed, a greater proportion of BOS‐ than placebo‐treated patients achieved complete dysphagia symptom resolution (“No” to Q2 for each daily DSQ diary entry in the 2 weeks before the study visit; i.e., a greater proportion of placebo‐ than BOS‐treated patients had dysphagia at each time point). Separation between treatment groups was observed as early as week 4 of therapy (week 4 = MPI 101‐06: 7.8% vs. 2.4%; SHP621‐301: 7.0% vs. 3.8%; Figure 2). Differences between the BOS and placebo groups were greatest at week 12 for MPI 101‐06 (17.7% vs. 9.5%; Δ: 0.11 [95% CI: −0.050, 0.254]) and week 8 for SHP621‐301 (11.3% vs. 4.8%; Δ: 0.06 [95% CI: −0.010, 0.123]). For BOS‐treated patients, the proportions of patients who achieved complete dysphagia symptom resolution increased at each time point up to week 12 of therapy (MPI 101‐06: week 4, 7.8%; week 8, 15.7%; week 12, 17.7%; SHP621‐301: week 4, 7.0%; week 8, 11.3%; week 12, 11.7%).

#### 3.2.3 Change From Baseline in the Number of Dysphagia‐Free Days

**FIGURE 3:**
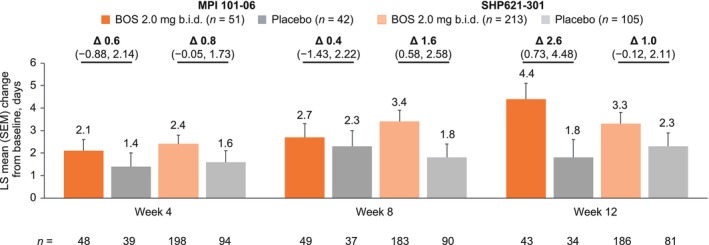
LS mean (SEM) change from baseline to weeks 4, 8, and 12 of therapy in the number of dysphagia‐free days (number of days with “No” to Q2 of the DSQ in the 2 weeks before a study visit) in patients who received up to 12 weeks of BOS 2.0 mg b.i.d. or placebo in MPI 101‐06 or SHP621‐301. Δ, difference; ANCOVA, analysis of covariance; b.i.d., twice daily; BOS, budesonide oral suspension; CI, confidence interval; DSQ, Dysphagia Symptom Questionnaire; LS, least‐squares; Q, question; SEM, standard error of the mean.

At baseline, the mean numbers of dysphagia‐free days (number of days with “No” to Q2 of the DSQ in the 2 weeks before the study visit) were similar for patients who received BOS or placebo (MPI 101‐06: 3.9 and 3.7 days, respectively; SHP621‐301: 3.6 and 3.7 days, respectively). BOS‐treated patients had greater LS mean improvements from baseline to weeks 4, 8, and 12 of therapy in the number of dysphagia‐free days than placebo‐treated patients (Figure 3). Differences in LS mean improvements in the number of dysphagia‐free days between the BOS and placebo groups were greatest at week 12 for MPI 101‐06 (+4.4 vs. +1.8 days; Δ: 2.6 [95% CI: 0.73, 4.48] days) and week 8 for SHP621‐301 (+3.4 vs. +1.8 days; Δ: 1.6 [95% CI: 0.58, 2.58] days).

#### 3.2.4 Dysphagia Symptoms but No Adaptive Behaviors or Pain

**FIGURE 4:**
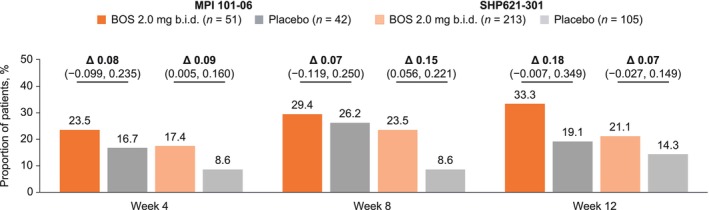
Proportion of patients who had dysphagia symptoms but no adaptive behaviors or pain (“Yes” to Q2 for at least one daily DSQ diary entry in the 2 weeks before a study visit but no daily score > 0 for Q3 and Q4) at weeks 4, 8, and 12 of therapy with BOS 2.0 mg b.i.d. or placebo in MPI 101‐06 or SHP621‐301. Δ values (presented in decimal form) are CMH stratum‐adjusted treatment differences in proportions of patients, representing differences after making groups comparable (adjusting) for age and dietary restriction. These Δ values may therefore differ from the raw percentage differences that are calculable from the proportions of patients presented for each treatment group. Δ, CMH‐adjusted difference; b.i.d., twice daily; BOS, budesonide oral suspension; CI, confidence interval; CMH, Cochran–Mantel–Haenszel; DSQ, Dysphagia Symptom Questionnaire; Q, question.

A greater proportion of BOS‐ than placebo‐treated patients reported dysphagia symptoms in the absence of adaptive behaviors and pain (“Yes” to Q2 for at least one daily DSQ diary entry in the 2 weeks before the study visit, but no daily score > 0 for Q3 or Q4) (Figure 4). The difference between BOS and placebo groups was most pronounced at week 12 for MPI 101‐06 (33.3% vs. 19.1%; Δ: 0.18 [95% CI: −0.007, 0.349]) and week 8 for SHP621‐301 (23.5% vs. 8.6%; Δ: 0.15 [95% CI: 0.056, 0.221]). The proportion of patients with dysphagia symptoms but no adaptive behaviors or pain was generally maintained from week 8 to 12 of therapy. During MPI 101‐06, the proportion of BOS‐treated patients who achieved this efficacy outcome increased at each time point from week 4 to 12 of therapy. During SHP621‐301, the proportion of BOS‐treated patients who achieved this efficacy outcome increased from week 4 to 8 and was generally maintained from week 8 to 12 of therapy.

#### 3.2.5 Post Hoc Outcomes Stratified by Esophageal Dilation History

Efficacy outcomes were generally unaffected by esophageal dilation history (Supporting Information). At most time points measured, a greater proportion of BOS‐ than placebo‐treated patients had complete dysphagia symptom resolution, or had dysphagia symptoms without adaptive behaviors or pain, irrespective of dilation history. Greater improvements from baseline in the number of dysphagia‐free days were also generally seen in BOS‐ than in placebo‐treated patients, irrespective of dilation history.

## 4 Discussion

These post hoc analyses of data from a phase 2 and a phase 3, double‐blind, placebo‐controlled clinical trial represent the first analyses of dysphagia symptom resolution outcomes for a swallowed corticosteroid approved by the FDA for the treatment of EoE [18]. We demonstrate that the time to first dysphagia symptom response was significantly shorter for patients who received BOS versus placebo, with separation between treatment groups observed from as early as week 2 of therapy. Dysphagia symptom resolution outcomes (complete dysphagia resolution, number of dysphagia‐free days, and dysphagia symptoms but no adaptive behaviors or pain) were improved over 12 weeks of therapy in BOS‐ versus placebo‐treated patients, with separation between treatment groups observed from week 4 of therapy (the earliest time point measured for these outcomes).

Despite the importance of comprehensive monitoring of both histology and symptoms in EoE [19], patients may be more likely to value tangible symptom relief over histologic improvement [17, 20, 21]. It is therefore important to consider how dysphagia symptom resolution and improvement (including swallowing symptoms) can be meaningfully evaluated in patients with EoE. A recent review by Hirano et al. proposed that the DSQ could be used to examine complete dysphagia symptom response to treatment and the number of dysphagia‐free days, both of which are clinically meaningful outcomes for patients with EoE [6, 20]. It has also been suggested that patients with EoE most value both short‐term and long‐term improvements in symptoms over other outcomes [17]. Thus, the speed of symptom response may be of importance in clinical practice. In our post hoc analyses, separation between BOS and placebo groups for the time to first dysphagia symptom response and for the proportion of patients who experienced complete dysphagia symptom resolution was observed at weeks 2 and 4 of therapy, respectively. Together, these results indicate rapid relief of dysphagia symptoms in patients receiving BOS. These findings are consistent with a phase 3 trial in patients receiving dupilumab 300 mg once weekly, in which separation between dupilumab‐ and placebo‐treated patients in the change from baseline in DSQ score was observed from as early as week 2 of therapy. While change from baseline in DSQ score was formally assessed only at week 24 in the dupilumab trial [11], our analyses assessed a percentage change response at 2‐week intervals up to week 12 of therapy.

Complete dysphagia symptom resolution (no dysphagia episodes over a 14‐day period, as captured in a patient‐reported electronic diary) has also been examined post hoc in a placebo‐controlled trial in adult patients with EoE who received 12 weeks of therapy with a fluticasone propionate orally disintegrating tablet 3.0 mg once daily [22]. Similar to our findings, a higher proportion of patients who received active treatment achieved complete dysphagia symptom resolution than those who received placebo (27% vs. 11%) [22]. Dysphagia symptom resolution was further evaluated over 6 weeks of therapy for budesonide orodispersible tablet (BOT) 1.0 mg (approved by the European Medicines Agency for adults aged > 18 years with EoE) [23, 24], using the unvalidated patient's global assessment (PatGA) of EoE activity numerical rating scale (score range: 0–10; higher scores indicate greater symptom severity) [25]. Compared with placebo recipients, a higher proportion of patients receiving BOT 1.0 mg b.i.d. achieved dysphagia symptom resolution (≤ 2 points on the scale; 64.4% vs. 24.1%) [24]. When comparing these findings, it is important to consider differences in the definitions of dysphagia symptom resolution, the treatment periods, the clinical trial designs, and the study populations [22, 24]. In particular, the dysphagia symptom resolution outcome examined in our analyses is highly stringent given that it uses an EoE‐specific, validated PRO measure [14]. This is in contrast to the outcome of minimal dysphagia (≤ 2 points on the PatGA of EoE scale) that was used during the BOT 1.0 mg b.i.d. clinical trial [24].

Compared with placebo, BOS‐treated patients in our analyses had greater LS mean improvements from baseline in the number of dysphagia‐free days up to week 12 of therapy. Phase 3 clinical trials are now increasingly using this metric and similar approaches to evaluate symptom improvement in response to treatments for EoE, showing improvements with active treatment versus placebo [6, 24, 26, 27, 28]. Reported methods include capturing the number of dysphagia days (number of days with “Yes” to Q2 in the DSQ, measured over 2 weeks) [28]; dysphagia‐free days (as captured by the DSQ) over a 28‐day period [27]; days with minimal dysphagia (≤ 2 points on the PatGA of EoE scale) [24]; and, in our post hoc analyses, dysphagia‐free days (“No” to Q2 of the DSQ in the 2 weeks before a study visit). The collective use of these outcomes demonstrates that the number of dysphagia days, or dysphagia‐free days, is considered a clinically meaningful measure of symptom improvement in patients with EoE. However, there remains an unmet need for alignment on how to measure symptom improvement in real‐world clinical practice, where validated questionnaires may not be routinely used.

The accuracy of dysphagia symptom assessment also represents an ongoing challenge in the field of EoE, because patients often develop adaptive eating behaviors to ease the passage of food, which may mask disease severity [29]. Dellon et al. have previously investigated the effect of BOS on dysphagia and pain (Q2 and Q4 of the DSQ) in MPI 101‐06, with no statistically significant difference observed in change from baseline in pain scores for BOS‐ versus placebo‐treated patients during the treatment period (weeks 4, 8, and 12 of therapy) [15]. Here, our analyses provided an alternative way to examine the effect of BOS on dysphagia and pain, first demonstrating that a higher proportion of BOS‐ than placebo‐treated patients experienced complete dysphagia symptom resolution at any time point. However, among those patients who did experience dysphagia symptoms (“Yes” to Q2 for at least one daily DSQ diary entry in the 2 weeks before the study visit), a greater proportion of BOS‐ than placebo‐treated patients had no adaptive behaviors or pain (no daily score > 0 for Q3 or Q4). This suggests that the severity of dysphagia was reduced in patients receiving BOS versus placebo because patients did not need to adapt their behaviors to manage swallowing (“No” to Q3) and did not experience any pain associated with dysphagia (“No” to Q4 [13, 14]), which would be potentially meaningful for patients.

Strengths of these analyses include that data were assessed from two large, robust, placebo‐controlled, randomized clinical trials. Dysphagia symptom resolution represents a disease activity metric that is increasingly being considered as a clinically meaningful measure of symptom improvement in patients with EoE. Dysphagia symptom resolution outcomes were evaluated using the DSQ, an EoE‐specific PRO that has been validated in patients aged ≥ 11 years and was developed in accordance with FDA guidelines to evaluate symptom outcomes in EoE [13, 14]. The Kaplan–Meier method was used to examine the time from baseline to the onset of first dysphagia symptom response at 2‐week intervals, representing a more comprehensive and frequent analysis of dysphagia symptom response than the prespecified analysis (in which patients completed the DSQ for each 2‐week period during the study, but dysphagia symptom response was formally evaluated only at week 12 of therapy) [8, 9, 30].

Limitations of our analyses include that the clinical trials were not powered for the post hoc efficacy outcomes and that these outcomes were only evaluated in patients who had non‐missing data for ≥ 8/14 days of the DSQ diary in the 2 weeks before a study visit.

Overall, the time to first dysphagia symptom response was significantly shorter for BOS‐ than placebo‐treated patients, with separation between treatment groups observed as early as the first time point measured, week 2 of therapy. At all time points, BOS‐treated patients experienced greater improvements in dysphagia symptom resolution outcomes over 12 weeks of therapy than placebo‐treated patients, with separation between treatment groups observed as early as week 4 of therapy (the earliest time point measured for these analyses). These findings support the conclusions from the prespecified analyses for MPI 101‐06 and SHP621‐301 and further demonstrate the efficacy of BOS 2.0 mg b.i.d. in managing dysphagia symptoms, and specifically the resolution of symptoms, in patients with EoE [8, 9]. Given the importance of tangible symptom relief to patients with EoE, there is a need to align on how dysphagia symptom resolution outcomes are evaluated, so that meaningful improvements in symptoms can be captured in real‐world clinical practice.

## Funding

These studies were funded by Meritage Pharma, Inc., now part of Shire, a member of the Takeda group of companies, and Shire ViroPharma, Inc., a member of the Takeda group of companies. These post hoc analyses were funded by Takeda Pharmaceuticals USA, Inc.

## Ethics Statement

These studies were performed in accordance with the Declaration of Helsinki, the Good Clinical Practice Guidelines, and the Consolidated Standards of Reporting Trials statement. The study protocols were approved by an institutional review board for each center.

## Consent

All patients or parents/caregivers provided written informed consent/assent before enrollment.

## Conflicts of Interest

E.S.D., D.A.K., V.A.M., and G.W.F. were involved directly with the MPI 101‐06 and SHP621‐301 clinical trials for BOS/Eohilia, which were funded by Meritage Pharma, Inc., now part of Shire, a member of the Takeda group of companies, and Shire ViroPharma, Inc., a member of the Takeda group of companies. E.S.D. has received research funding from Adare Pharmaceuticals/Ellodi Pharmaceuticals, Allakos, Arena Pharmaceuticals/Pfizer, AstraZeneca, Celgene/Receptos/Bristol Myers Squibb, Celldex Therapeutics, EuPRAXIA, Ferring Pharmaceuticals, GSK, Meritage Pharma, Inc., Miraca Life Sciences, Nutricia, Regeneron Pharmaceuticals, Revolo Biotherapeutics, Sanofi, and Shire, a Takeda company; is a consultant for AbbVie, Adare Pharmaceuticals/Ellodi Pharmaceuticals, Akeso Biopharma, Alfasigma Global, ALK, Allakos, Amgen, Apollo, Aqilion AB, Arena Pharmaceuticals/Pfizer, ASLAN Pharmaceuticals, AstraZeneca, Avir Pharma, BioCryst Pharmaceuticals, Bryn Pharma, Calypso Biotech, Celgene/Receptos/Bristol Myers Squibb, Celldex Therapeutics, Dr. Falk Pharma, EsoCap Biotech, EuPRAXIA, Ferring Pharmaceuticals, GI Reviewers, GSK, Holoclara, Inc., Invea Therapeutics, Knightpoint, Lucid Diagnostics, Morphic Therapeutic, Nexstone Immunology/Uniquity Bio, Nutricia, Parexel/Calyx Clinical Trial Solutions, Phathom Pharmaceuticals, Regeneron Pharmaceuticals, Revolo Biotherapeutics, Robarts Clinical Trials, Inc./Alimentiv, Inc., Sanofi, Shire, a Takeda company, Target RWE, and Upstream Bio; and has received educational grants from Allakos, Aqilion AB, Holoclara, Inc., and Invea Therapeutics. D.A.K. has received research funding from Shire, a Takeda company, and has provided consultancy for Celgene/Receptos/Bristol Myers Squibb. V.A.M. has received research funding from Meritage Pharma, Inc., and Shire, a Takeda company; serves on an adjudication committee for Alladapt Immunotherapeutics; and is a consultant for Allakos, Phathom Pharmaceuticals, Regeneron Pharmaceuticals, Sanofi/Genzyme, and Shire, a Takeda company. G.W.F. has received research funding from Adare Pharmaceuticals/Ellodi Pharmaceuticals, Allakos, Arena Pharmaceuticals/Pfizer, Celgene/Receptos/Bristol Myers Squibb, Lucid Diagnostics, NexEos Bio, Regeneron Pharmaceuticals, Sanofi, and Shire, a Takeda company; and is a consultant for Adare Pharmaceuticals/Ellodi Pharmaceuticals, Allakos, Celgene/Receptos/Bristol Myers Squibb, Lucid Diagnostics, Nexstone Immunology/Uniquity Bio, Phathom Pharmaceuticals, Sanofi, Shire, a Takeda company, and Upstream Bio. M.J.T. is an employee of Takeda Development Center Americas, Inc., and a stockholder of Takeda Pharmaceutical Company Limited. P.C.G. and B.T. are employees of Takeda Pharmaceuticals USA, Inc., and stockholders of Takeda Pharmaceutical Company Limited. J.B. was an employee of Takeda Development Center Americas, Inc. at the time that the analyses for the MPI 101‐06 and SHP621‐301 studies were conducted.

## Supporting information




**Figure S1:** CONSORT flow diagram for MPI 101‐06 and SHP621‐301.
**Table S1:** Effect of prior esophageal dilation on post hoc dysphagia symptom resolution outcomes assessed at weeks 4, 8, and 12 of therapy for patients who received up to 12 weeks of BOS 2.0 mg b.i.d. or placebo in MPI 101‐06.
**Table S2:** Effect of prior esophageal dilation on post hoc dysphagia symptom resolution outcomes assessed at weeks 4, 8, and 12 of therapy for patients who received up to 12 weeks of BOS 2.0 mg b.i.d. or placebo in SHP621‐301

## Data Availability

The data sets, including the redacted study protocol, redacted statistical analysis plan, and individual participants' data supporting the results reported in this article, will be made available within 3 months from the initial request to researchers who provide a methodologically sound proposal. The data will be provided after its de‐identification, in compliance with applicable privacy laws, data protection, and requirements for consent and anonymization.
