# Supplementary material for: Effect of Budesonide Oral Suspension on Time to First Dysphagia Symptom Response and Dysphagia Symptom Resolution Outcomes in Patients With Eosinophilic Esophagitis
Source: J Gastroenterol Hepatol. 2026 Feb 19;41(3):927–36. doi: 10.1111/jgh.70205 (PMC12969252; doi:10.1111/jgh.70205)
Supplement: Supplementary file 1 — Figure S1: CONSORT flow diagram for MPI 101‐06 and SHP621‐301. Table S1: Effect of prior esophageal dilation on post hoc dysphagia symptom resolution outcomes assessed at weeks 4, 8, and 12 of therapy for patients who received up to 12 weeks of BOS 2.0 mg b.i.d. or placebo in MPI 101‐06. Table S2: Effect of prior esophageal dilation on post hoc dysphagia symptom resolution outcomes assessed at weeks 4, 8, and 12 of therapy for patients who received up to 12 weeks of BOS 2.0 mg b.i.d. or placebo in SHP621‐301 [file JGH-41-927-s001.docx]

**Supporting Information**

**Effect of budesonide oral suspension on time to first dysphagia symptom response and dysphagia symptom resolution outcomes in patients with eosinophilic esophagitis**

**Supplemental Methods**

***Key inclusion and exclusion criteria for MPI 101-06 and SHP621-301***

For both clinical trials, patients were eligible for inclusion if they had histologic evidence of eosinophilic esophagitis (EoE; ≥15 eosinophils per high-power field in at least two levels of the esophagus), had dysphagia symptoms for ≥4 days, and had completed the Dysphagia Symptom Questionnaire (DSQ)^1^ on ≥70% of days in any consecutive 2-week period during the 3–6-week screening period.^2, 3^ In addition, patients were required to have proton pump inhibitor-unresponsive EoE, as determined by a high-dose proton pump inhibitor trial, for ≥ 6–8 weeks before trial entry (as per diagnostic criteria at the time of trial design^4, 5^), and were required to be willing to continue with any dietary, environmental, or medical therapy that was in effect at screening.^2, 3, 6, 7^ Patients were excluded from the clinical trials if they had undergone esophageal dilation ≤12 months before enrollment (MPI 101-06) or ≤3 months before screening (SHP621-301), presented with a high-grade esophageal stricture at the screening endoscopy, or had used swallowed topical corticosteroids for EoE or systemic corticosteroids for any condition ≤4 weeks before the screening esophagogastroduodenoscopy (EGD; MPI 101-06) or between the screening EGD and baseline (SHP621-301). Patients were also excluded if they had used an immunomodulatory therapy ≤8 weeks before the screening EGD (MPI 101-06),^2^ anticipated immunomodulatory therapy use during the trial period (SHP621-301), or had been on a pure liquid diet or six-food elimination diet (SHP621-301).^3^ Full descriptions of inclusion and exclusion criteria for both clinical trials are reported elsewhere.^2, 3^

***Overview of the DSQ***

Questions (Qs) in the DSQ evaluate the frequency and severity of dysphagia. Patients must have eaten solid food (Q1) to proceed with the questionnaire.^1^ Evaluated clinical concepts are listed in **Table 1**.

The scoring algorithm for the DSQ was constructed using responses to Q2 and Q3 (frequency and severity of dysphagia, respectively) to generate a combined DSQ score. Scores can range from 0 to 84, with higher values indicating greater severity of dysphagia. A minimum of eight entries in a 14-day period are required to generate a combined DSQ score.^8^ Q4 (pain when swallowing food) was a standalone item that was scored separately. Scores could range from 0 to 56, with higher scores indicating greater pain.^8^

**Post hoc *outcomes, stratified by esophageal dilation history***

Data for each of the *post hoc* outcomes were stratified by reported dilation history (esophageal dilation >12 months before enrollment [MPI 101-06] or >3 months before screening [SHP621-301]) or no reported dilation history (no prior dilation).^2, 3^ When available, esophageal dilation history was determined from patient self-report or medical records.

**Supplemental Results**

***Post hoc* *outcomes, stratified by esophageal dilation history***

In MPI 101-06, 17 patients had a reported dilation history (BOS, *n* = 10; placebo, *n* = 7) and 76 patients had no reported dilation history (BOS, *n* = 41; placebo, *n* = 35) (**Table S1**). In SHP621-301, 136 patients had a reported dilation history (BOS, *n* = 91; placebo, *n* = 45) and 182 patients had no reported dilation history (BOS, *n* = 122; placebo, *n* = 60) (**Table S2**).

*Complete dysphagia symptom resolution*

At most time points measured, a greater proportion of BOS- than placebo-treated patients achieved complete dysphagia symptom resolution (‘No’ to Q2 for each daily DSQ diary entry in the 2 weeks before the study visit) in both MPI 101-06 and SHP621-301, irrespective of dilation history (**Tables S1 and S2**). Exceptions were observed at week 12 in both trials, when no BOS- or placebo-treated patients with a history of dilation in MPI 101-06 had complete dysphagia symptom resolution, and a similar proportion of BOS- and placebo-treated patients had complete dysphagia symptom resolution in the no dilation history group in SHP621-301. In general, and irrespective of treatment, more patients in the no dilation history groups achieved this efficacy outcome than those in the dilation history groups in both trials.

*Change from baseline in the number of dysphagia-free days*

In general, BOS-treated patients had greater least-squares mean improvements from baseline to weeks 4, 8, and 12 of therapy in the number of dysphagia-free days (number of days with ‘No’ to Q2 in the DSQ in the 2 weeks before the study visit) than placebo-treated patients, irrespective of dilation history (**Table S1 and S2**). Greater least-squares mean improvements from baseline were observed at each time point in BOS-treated patients in the dilation history group than in the no dilation history group in MPI 101-06, whereas the opposite was observed in SHP621-301.

During MPI 101-06, the number of dysphagia-free days in BOS-treated patients increased at each time point from week 4 to 12 of therapy, but not for placebo-treated patients. In the no dilation history group, the number of dysphagia-free days was slightly higher at week 8 of therapy in placebo- than BOS-treated patients.

During SHP621-301, the number of dysphagia-free days in BOS-treated patients increased from week 4 to 8 of therapy and was generally maintained from week 8 to 12 of therapy, irrespective of dilation history. No difference was observed between BOS- and placebo-treated patients in the dilation history group at week 12 of therapy.

*Dysphagia symptoms but no adaptive behaviors or pain*

At almost all time points measured, a greater proportion of BOS- than placebo-treated patients reported dysphagia symptoms but no adaptive behaviors or pain (‘Yes’ to Q2 for at least one daily DSQ diary entry in the 2 weeks before the study visit but no daily score >0 for Q3 and Q4), irrespective of dilation history (**Tables S1 and S2**). An exception was observed at week 8 for the no dilation history group in MPI 101-06, where a slightly lower proportion of BOS- than placebo-treated patients achieved this outcome.

During MPI 101-06, the proportion of BOS-treated patients who achieved this efficacy outcome in the dilation history group increased at each time point from week 4 to 8 of therapy and was generally maintained from week 8 to 12 of therapy. In the no dilation history group, the proportion of BOS-treated patients who achieved this efficacy outcome increased at each time point from week 4 to 12 of therapy. A greater proportion of BOS-treated patients in the no dilation history group than in the dilation history group achieved this outcome at weeks 4 and 12 of therapy, whereas the inverse was observed at week 8 of therapy.

During SHP621-301, the proportion of BOS-treated patients who reported dysphagia symptoms but no adaptive behaviors or pain increased at each time point from week 4 to 8 of therapy and was generally maintained from week 8 to 12 of therapy, irrespective of dilation history. The proportions of BOS-treated patients who achieved this outcome were similar at week 4 of therapy in the dilation history groups and were higher at weeks 8 and 12 of therapy in the dilation history group than in the no dilation history group.

**Figure S1** CONSORT flow diagram for MPI 101-06 and SHP621-301.


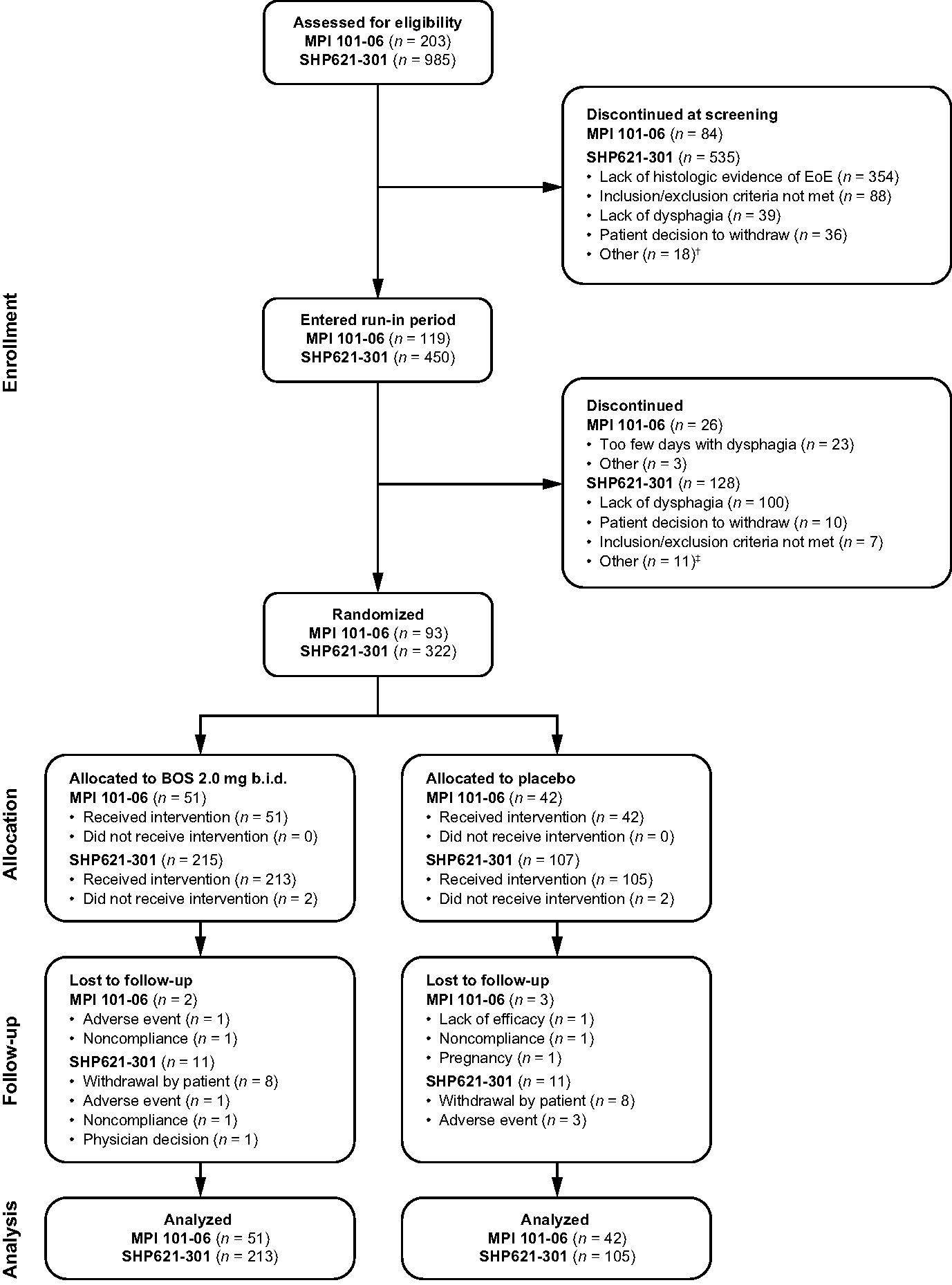


^†^Other reasons for study discontinuation were: physician decision to withdraw (*n* = 9); lost to follow-up (*n* = 4); parent/guardian decision to withdraw (*n* = 2); adverse event (*n* = 1); pregnancy (*n* = 1); and protocol deviation (*n* = 1). ^‡^Other reasons for study discontinuation were: lost to follow-up (*n* = 4); lack of adherence to study treatment (*n* = 4); lack of histologic evidence of EoE (*n* = 2); and adverse event (*n* = 1).

b.i.d., twice daily; BOS, budesonide oral suspension; CONSORT, Consolidated Standards of Reporting Trials; DSQ, Dysphagia Symptom Questionnaire; EGD, esophagogastroduodenoscopy; EoE, eosinophilic esophagitis; ICF, informed consent form.

**Table S1** Effect of prior esophageal dilation on *post hoc* dysphagia symptom resolution outcomes assessed at weeks 4, 8, and 12 of therapy for patients who received up to 12 weeks of BOS 2.0 mg b.i.d. or placebo in MPI 101-06

| **Efficacy outcome** | **Dilation history** | | | | | | **No dilation history** | | | | | |
| --- | --- | --- | --- | --- | --- | --- | --- | --- | --- | --- | --- | --- |
|  | **Week 4** | | **Week 8** | | **Week 12** | | **Week 4** | | **Week 8** | | **Week 12** | |
|  | **BOS**  **2.0 mg b.i.d.**  **(*n* = 10)** | **Placebo**  **(*n* = 7)** | **BOS**  **2.0 mg b.i.d.**  **(*n* = 10)** | **Placebo**  **(*n* = 7)** | **BOS**  **2.0 mg b.i.d.**  **(*n* = 10)** | **Placebo**  **(*n* = 7)** | **BOS**  **2.0 mg b.i.d.**  **(*n* = 41)** | **Placebo**  **(*n* = 35)** | **BOS**  **2.0 mg b.i.d.**  **(*n* = 41)** | **Placebo**  **(*n* = 35)** | **BOS**  **2.0 mg b.i.d.**  **(*n* = 41)** | **Placebo**  **(*n* = 35)** |
| **Complete dysphagia symptom resolution, *n* (%)^†,‡^** | 1 (10.0) | 0 (0.0) | 1 (10.0) | 0 (0.0) | 0 (0.0) | 0 (0.0) | 3 (7.3) | 1 (2.9) | 7 (17.1) | 5 (14.3) | 9 (22.0) | 4 (11.4) |
| Δ,  (95% CI) | 0.10  (−0.308, 0.445) | | 0.10  (−0.308, 0.445) | | N/A | | 0.04  (−0.089, 0.174) | | 0.04  (−0.144, 0.210) | | 0.13  (−0.056, 0.301) | |
| **Change from baseline in number of dysphagia-free days, LS mean (SEM)^§^** | 2.3  (0.87) | −0.5 (1.01) | 4.1 (0.86) | 0.9 (1.17) | 6.2  (0.94) | −1.5 (1.21) | 2.0 (0.62) | 1.8 (0.72) | 2.5 (0.72) | 2.7 (0.88) | 4.2 (0.74) | 2.4 (0.87) |
| Δ,  (95% CI) | 2.8  (−0.09, 5.76) | | 3.2  (−0.11, 6.52) | | 7.7  (4.10, 11.30) | | 0.2  (−1.59, 1.93) | | −0.3  (−2.38, 1.86) | | 1.8  (−0.33, 3.94) | |
| **Dysphagia symptoms but no adaptive behaviors or pain, *n* (%)**^¶^**^,‡^** | 2 (20.0) | 1  (14.3) | 4 (40.0) | 1  (14.3) | 2 (20.0) | 0 (0.0) | 10 (24.4) | 6  (17.1) | 11 (26.8) | 10 (28.6) | 15 (36.6) | 8  (22.9) |
| Δ,  (95% CI) | 0.06  (−0.410, 0.471) | | 0.26  (−0.263, 0.647) | | 0.20  (−0.263, 0.566) | | 0.08  (−0.119, 0.255) | | 0.02  (−0.184, 0.226) | | 0.18  (−0.036, 0.369) | |

^†^‘No’ to Q2 for each daily DSQ diary entry in the 2 weeks before the study visit.

^‡^Δ values (presented in decimal form) are CMH stratum-adjusted treatment differences in proportions of patients, representing differences after making groups comparable (adjusting) for age and dietary restriction. These Δ values may therefore differ from the raw percentage differences that are calculable from the proportions of patients presented for each treatment group.

^§^Number of days with ‘No’ to Q2 in the DSQ in the 2 weeks before the study visit.

^¶^‘Yes’ to Q2 for at least one daily DSQ diary entry in the 2 weeks before a study visit but no daily score >0 for Q3 and Q4.

Δ, CMH-adjusted difference; b.i.d., twice daily; BOS, budesonide oral suspension; CI, confidence interval; CMH, Cochran–Mantel–Haenszel; DSQ, Dysphagia Symptom Questionnaire; EoE, eosinophilic esophagitis; LS, least-squares; N/A, not applicable; Q, question; SEM, standard error of the mean.

**Table S2** Effect of prior esophageal dilation on *post hoc* dysphagia symptom resolution outcomes assessed at weeks 4, 8, and 12 of therapy for patients who received up to 12 weeks of BOS 2.0 mg b.i.d. or placebo in SHP621-301

| **Efficacy outcome** | **Dilation history** | | | | | | **No dilation history** | | | | | |
| --- | --- | --- | --- | --- | --- | --- | --- | --- | --- | --- | --- | --- |
|  | **Week 4** | | **Week 8** | | **Week 12** | | **Week 4** | | **Week 8** | | **Week 12** | |
|  | **BOS**  **2.0 mg b.i.d.**  **(*n* = 91)** | **Placebo**  **(*n* = 45)** | **BOS**  **2.0 mg b.i.d.**  **(*n* = 91)** | **Placebo**  **(*n* = 45)** | **BOS**  **2.0 mg b.i.d.**  **(*n* = 91)** | **Placebo**  **(*n* = 45)** | **BOS**  **2.0 mg b.i.d.**  **(*n* = 122)** | **Placebo**  **(*n* = 60)** | **BOS**  **2.0 mg b.i.d.**  **(*n* = 122)** | **Placebo**  **(*n* = 60)** | **BOS**  **2.0 mg b.i.d.**  **(*n* = 122)** | **Placebo**  **(*n* = 60)** |
| **Complete dysphagia symptom resolution, *n* (%)^†,‡^** | 4 (4.4) | 1 (2.2) | 10 (11.0) | 2 (4.4) | 13 (14.3) | 3 (6.7) | 11 (9.0) | 3 (5.0) | 14 (11.5) | 3 (5.0) | 12 (9.8) | 6 (10.0) |
| Δ,  (95% CI) | 0.02  (−0.079, 0.093) | | 0.07  (−0.051, 0.159) | | 0.07  (−0.058, 0.172) | | 0.04  (−0.058, 0.118) | | 0.06  (−0.050, 0.138) | | −0.01  (−0.117, 0.082) | |
| **Change from baseline in number of dysphagia-free days, LS mean (SEM)^§^** | 1.4  (0.59) | 1.2 (0.80) | 2.7 (0.68) | 2.1 (0.93) | 2.3  (0.77) | 2.3 (1.10) | 2.9 (0.52) | 1.6 (0.57) | 3.6 (0.58) | 1.4 (0.63) | 3.5 (0.62) | 1.9 (0.70) |
| Δ,  (95% CI) | 0.2  (−1.20, 1.61) | | 0.7  (−1.00, 2.31) | | 0.0  (−1.86, 1.87) | | 1.3  (0.16, 2.48) | | 2.1  (0.86, 3.41) | | 1.7  (0.25, 3.07) | |
| **Dysphagia symptoms but no adaptive behaviors or pain, *n* (%)**^¶^**^,‡^** | 16 (17.6) | 6  (13.3) | 24 (26.4) | 3  (6.7) | 24 (26.4) | 8 (17.8) | 21 (17.2) | 3  (5.0) | 26 (21.3) | 6  (10.0) | 21 (17.2) | 7 (11.7) |
| Δ,  (95% CI) | 0.05  (−0.096, 0.172) | | 0.20  (0.053, 0.308) | | 0.09  (−0.074, 0.220) | | 0.12  (0.019, 0.207) | | 0.10  (−0.029, 0.199) | | 0.05  (−0.075, 0.146) | |

^†^‘No’ to Q2 for each daily DSQ diary entry in the 2 weeks before the study visit.

^‡^Δ values (presented in decimal form) are CMH stratum-adjusted treatment differences in proportions of patients, representing differences after making groups comparable (adjusting) for age and dietary restriction. These Δ values may therefore differ from the raw percentage differences that are calculable from the proportions of patients presented for each treatment group.

^§^Number of days with ‘No’ to Q2 in the DSQ in the 2 weeks before the study visit.

^¶^‘Yes’ to Q2 for at least one daily DSQ diary entry in the 2 weeks before the study visit but no daily score >0 for Q3 and Q4.

Δ, CMH-adjusted difference; b.i.d., twice daily; BOS, budesonide oral suspension; CI, confidence interval; CMH, Cochran–Mantel–Haenszel; DSQ, Dysphagia Symptom Questionnaire; EoE, eosinophilic esophagitis; LS, least-squares; Q, question; SEM, standard error of the mean.

**References**

[1] Dellon ES, Irani AM, Hill MR, Hirano I. Development and field testing of a novel patient-reported outcome measure of dysphagia in patients with eosinophilic esophagitis. *Aliment. Pharmacol. Ther.* 2013; **38**: 634–42.

[2] Dellon ES, Katzka DA, Collins MH *et al.* Budesonide oral suspension improves symptomatic, endoscopic, and histologic parameters compared with placebo in patients with eosinophilic esophagitis. *Gastroenterology*. 2017; **152**: 776–86 e5.

[3] Hirano I, Collins MH, Katzka DA *et al.* Budesonide oral suspension improves outcomes in patients with eosinophilic esophagitis: results from a phase 3 trial. *Clin. Gastroenterol. Hepatol.* 2022; **20**: 525–34 e10.

[4] Liacouras CA, Furuta GT, Hirano I *et al.* Eosinophilic esophagitis: updated consensus recommendations for children and adults. *J. Allergy Clin. Immunol.* 2011; **128**: 3–22.

[5] Dellon ES, Gonsalves N, Hirano I *et al.* ACG clinical guideline: evidenced based approach to the diagnosis and management of esophageal eosinophilia and eosinophilic esophagitis (EoE). *Am J Gastroenterol*. 2013; **108**: 679–92; quiz 93.

[6] ClinicalTrials.gov. OBS in adolescent and adults with EoE: a phase II, randomized, double-blind, placebo controlled, study with an open label extension. Available from URL: <https://clinicaltrials.gov/study/NCT01642212>.

[7] ClinicalTrials.gov. A study in adolescents and adults with eosinophilic esophagitis (EoE) measuring histologic response and determine if reduction in dysphagia is achieved. Available from URL: <https://clinicaltrials.gov/study/NCT02605837>.

[8] Hudgens S, Evans C, Phillips E, Hill M. Psychometric validation of the Dysphagia Symptom Questionnaire in patients with eosinophilic esophagitis treated with budesonide oral suspension. *J. Patient Rep. Outcomes*. 2017; **1**: 3.
